# Chilly Churches

**Published:** 1897-10-23

**Authors:** 


### Editor's Letter-Box.

[Our Correspondents are reminded that prolixity is a great bar to publication, and that brevity of style and conciseness of statement greatly facilitate early insertion.]

### CHILLY CHURCHES.

A medical man, who signs himself "Disgusted," writes: Under the above heading you say that doctors have none too good a reputation in regard to forms and ceremonies, and which I believe is quite correct; but I wish to point out to you another cause in addition to the one you give, *i.e.*, chilly churches, for medical men being set against church-going. I refer to the manner in which clergymen and ministers of religion insult and oppose the medical profession by encouraging quackery and all sorts of medical heresy. I enclose you an advertisement from a Warwickshire paper bearing on this point. The question is, How are medical men to retaliate? The only way I know is by keeping away from church. How would the clergy like the medical profession to openly oppose them and publicly give support to every idea that would rob them of their livelihood? If the present state of affairs continues to exist, medical men must openly wage war against the teachers of religion, and perhaps then the clergy will learn to devote more time to parish work and preparation of sermons and less to other people's business.

*** With the above letter is enclosed an advertisement of a well-known quack, containing the names of many clerics of many denominations, which purport to be their signatures. So is religion dragged through the mire by those who profess to be its ministers.—Ed. *T.H.*

